# Successful Identification and Treatment of Cancer of Unknown Primary Originating From Gastric Cancer Using Comprehensive Genomic Profiling and Immune Checkpoint Inhibitor Therapy: A Case Report

**DOI:** 10.1002/cnr2.70338

**Published:** 2025-09-08

**Authors:** Takahiro Sasaki, Sayaka Yuzawa, Hiroki Tanabe, Yusuke Ono, Keitaro Takahashi, Katsuyoshi Ando, Nobuhiro Ueno, Shin Kashima, Kentaro Moriichi, Mishie Tanino, Yusuke Mizukami, Mikihiro Fujiya

**Affiliations:** ^1^ Division of Gastroenterology, Department of Internal Medicine Asahikawa Medical University Asahikawa Japan; ^2^ Department of Diagnostic Pathology, Asahikawa Medical University Hospital Asahikawa Medical University Asahikawa Japan; ^3^ Oncology Center Asahikawa Medical University Hospital Asahikawa Japan; ^4^ Institute of Biomedical Research Sapporo‐Higashi Tokushukai Hospital Sapporo Japan

**Keywords:** CGP, CUP, ICI

## Abstract

**Background:**

Cancer of unknown primary (CUP) is a challenging malignancy characterized by metastatic tumors with an unidentified primary site, even after extensive pathological and radiographic evaluation. Recent advancements in gene expression profiling and comprehensive genomic profiling (CGP) using next‐generation sequencing (NGS) have enabled the identification of potential tissue origins, thereby facilitating personalized treatment strategies. Although most cases of CUP present as adenocarcinomas or poorly differentiated tumors, the treatment remains largely empirical, with limited success from molecularly tailored therapies. However, advances in tumor DNA sequencing and targeted therapies hold great promise for enhancing patient outcomes.

**Case:**

A 72‐year‐old woman presented with epigastric pain and was diagnosed with a duodenal tumor and gastric ulceration via esophagogastroduodenoscopy. A histological evaluation revealed poorly differentiated adenocarcinoma in the duodenum, and the immunohistochemistry findings supported a pancreatobiliary origin. An endoscopic ultrasound‐guided biopsy confirmed poorly differentiated adenocarcinoma in the duodenum, while a subsequent gastric examination revealed well‐differentiated adenocarcinoma, suggesting dual malignancies. The patient underwent neoadjuvant chemotherapy, followed by pancreatoduodenectomy with distal gastrectomy. The CUP was staged as poorly differentiated adenocarcinoma (pStage IVB), while the gastric cancer was staged as well‐differentiated adenocarcinoma (pStage IA). Despite adjuvant TS‐1 therapy, lymph node metastasis near the superior mesenteric artery continued to progress. CGP revealed high microsatellite instability and a high tumor mutational burden, along with multiple actionable genetic mutations. Pembrolizumab monotherapy was initiated, leading to complete remission, with no recurrence observed at 1 year after treatment cessation. Genetic and immunohistochemical investigations have identified microsatellite instability in both CUP and gastric cancer tissues, suggesting a shared origin. Targeted gene sequencing confirmed common genetic variations, ultimately revealing that the CUP originated from gastric cancer cells.

**Conclusion:**

This case highlights the critical role of CGP in the diagnosis and treatment of CUP. The use of advanced molecular techniques, including NGS, revealed the gastric origin of CUP and identified actionable biomarkers, leading to successful treatment with immune checkpoint inhibitors.

## 1 Introduction

Cancer of unknown primary (CUP) is defined as a histologically confirmed malignancy without an established primary site after pathological evaluation and radiographic examinations [1]. It is a complex and challenging condition where one or more metastatic tumors are present, but the location of the original tumor is unknown. This may be due to the primary cancer being undetectable by current imaging methods or having regressed by the time metastatic tumors are discovered. The diagnostic process begins with a thorough clinical evaluation, which includes a detailed patient history and physical examination. This is followed by imaging studies to locate and characterize metastases. Common imaging modalities include computed tomography (CT), magnetic resonance imaging, and positron emission tomography‐CT. Despite these techniques, the primary cancer is often not visualized, which complicates the diagnosis and treatment planning. CUP accounted for < 5% of all cancers. With the advent of gene expression profiling and comprehensive genomic profiling (CGP), 1%–2% of new cancer diagnoses are designated as CUP [2]. Gene expression profiling and next‐generation sequencing (NGS) may identify a potential site of origin in patients with CUP. Specific gene expression profiles have been well recognized in most cancers according to their site of origin, which reflects the different expression profiles present in their normal tissues of origin [3, 4]. The National Cancer Institute recommends routine testing to investigate gene expression levels and epigenetic factors [5]. Over the past decade, CGP has been developed to predict the tumor site of origins in patients with CUP.

Relative to patients with known primary tumors, the prognosis for patients with CUP is unfavorable, with a median overall survival rate of < 12 months [6]. The initial presentation of CUP typically involves symptoms related to metastatic spread, rather than the primary tumor. Common sites for metastases include the lymph nodes, liver, lungs, peritoneum, and bones. The symptoms experienced depend on the location and extent of metastasis but may include weight loss, pain, and general fatigue. Because the primary tumor is not initially detected, treatment is often empirical and based on the manifestations of metastatic disease. Most CUP cases are adenocarcinomas, which are often poorly differentiated or undifferentiated tumors. There are less frequent instances of squamous cell carcinomas and neuroendocrine tumors. Trials comparing empirical chemotherapy with molecularly tailored therapies have not demonstrated significant improvements in patient outcomes. Additionally, it is difficult to select an appropriate systemic therapy to match a specific type of cancer. Therefore, a molecular diagnosis that identifies the origin is expected to inform treatment selection and improve the prognosis. However, mutation profiling alone is not sufficient to guide the personalized treatment, given that targeted therapies against a particular driver mutation can act differently in different tumor types. This is mainly due to insufficient evidence supporting the improvement in the prognosis of CUP managed by the approach. With the advent of NGS technology, massively parallel sequencing of tumor DNA offers great opportunities to identify actionable variations and enables targeted therapy in oncologic practice. Effective treatment strategies for CUP may benefit from a combination of molecular profiling and traditional clinical approaches to more accurately tailor therapies and improve patient outcomes [7].

We herein report the case of a patient with CUP in which a precise diagnosis of gastric cancer origin enabled personalized treatment with targeted therapy, based on a comprehensive genomic analysis. The patient received immune checkpoint inhibitor (ICI) monotherapy and has achieved a complete response.

## 2 Case

**FIGURE 1:**
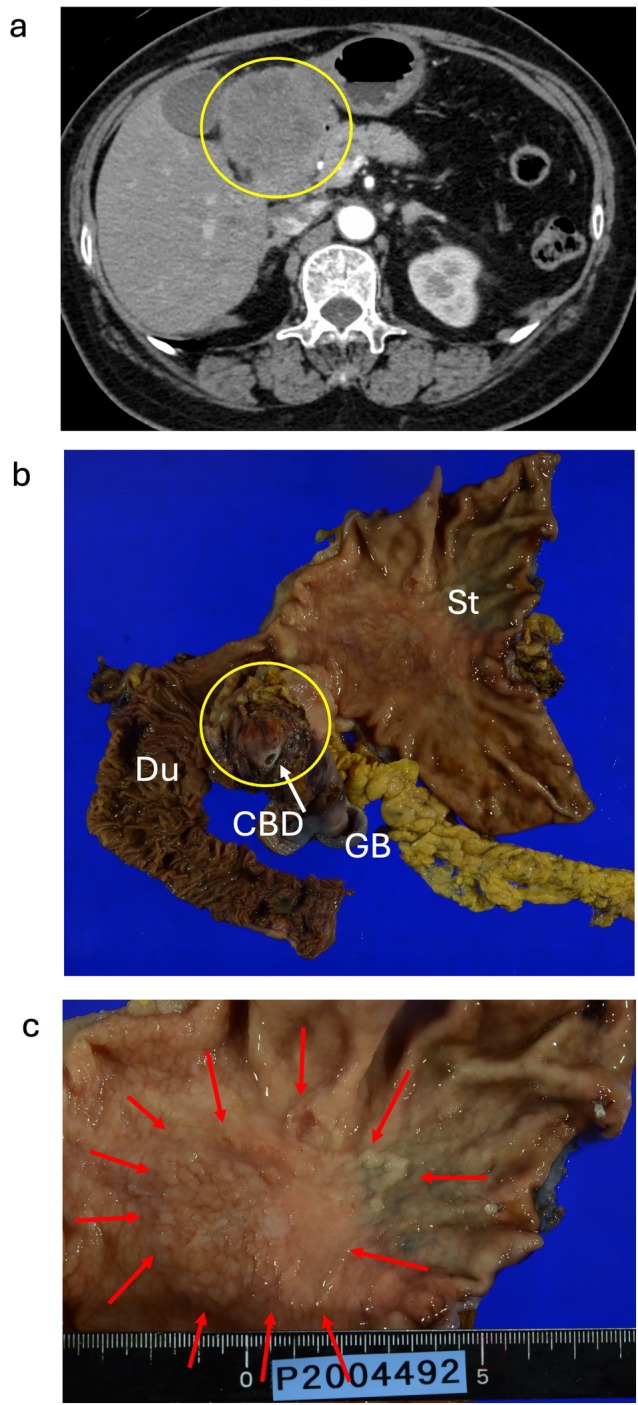
Cancer of unknown primary (CUP) and early gastric cancer. (a) A computed tomographic image of CUP shows a heterogeneous density mass at the portal hepatis, growing to the gall bladder and the pancreatic head. Yellow circle indicates the main tumor. (b) Surgically resected CUP tissue. The main tumor involves to the duodenum (Du), gall bladder (GB), and common bile duct (CBD). The tumor is separated from the stomach (St). Yellow circle indicates the main tumor. (c) Early gastric cancer forms a laterally spreading tumor. Red arrows indicate the lateral margin.

**FIGURE 2:**
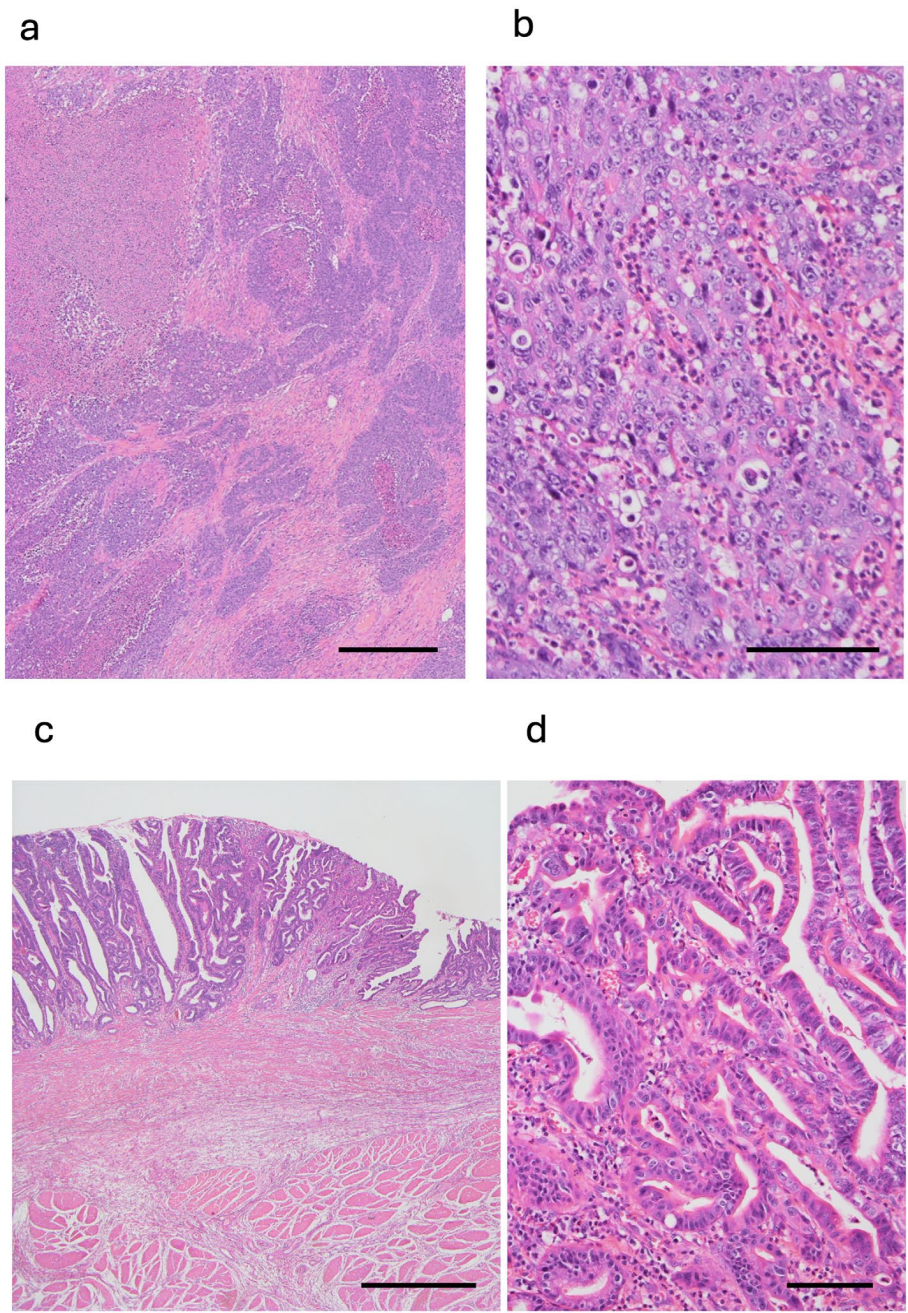
Histological findings of the carcinomas. (a) Poorly differentiated adenocarcinoma in the cancer of unknown primary (CUP) presents as a solid and cribriform structure with marked necrosis. (b) A high‐power view of the CUP shows poorly differentiated cancer cells with pleomorphic nuclei. (c) The mucosal lesion of gastric carcinoma shows mild lymphocytic infiltration. (d) A high‐power view shows well‐differentiated tubular adenocarcinoma. Scale bars: (a) 500 μm, (b) 100 μm, (c) 1 mm, (d) 100 μm.

**FIGURE 3:**
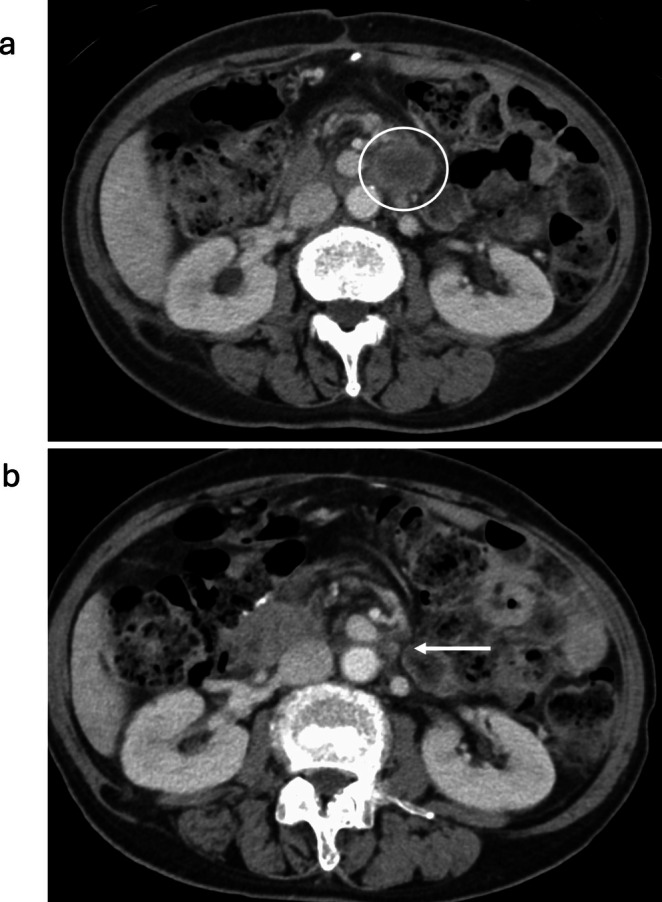
Recurred paraaortic lymph node metastasis. (a) A lymph node of 4 cm in diameter was observed 5 months after surgery (white circle). (b) The lymph node shrunk to < 1 cm after pembrolizumab treatment (white arrow).

**FIGURE 4:**
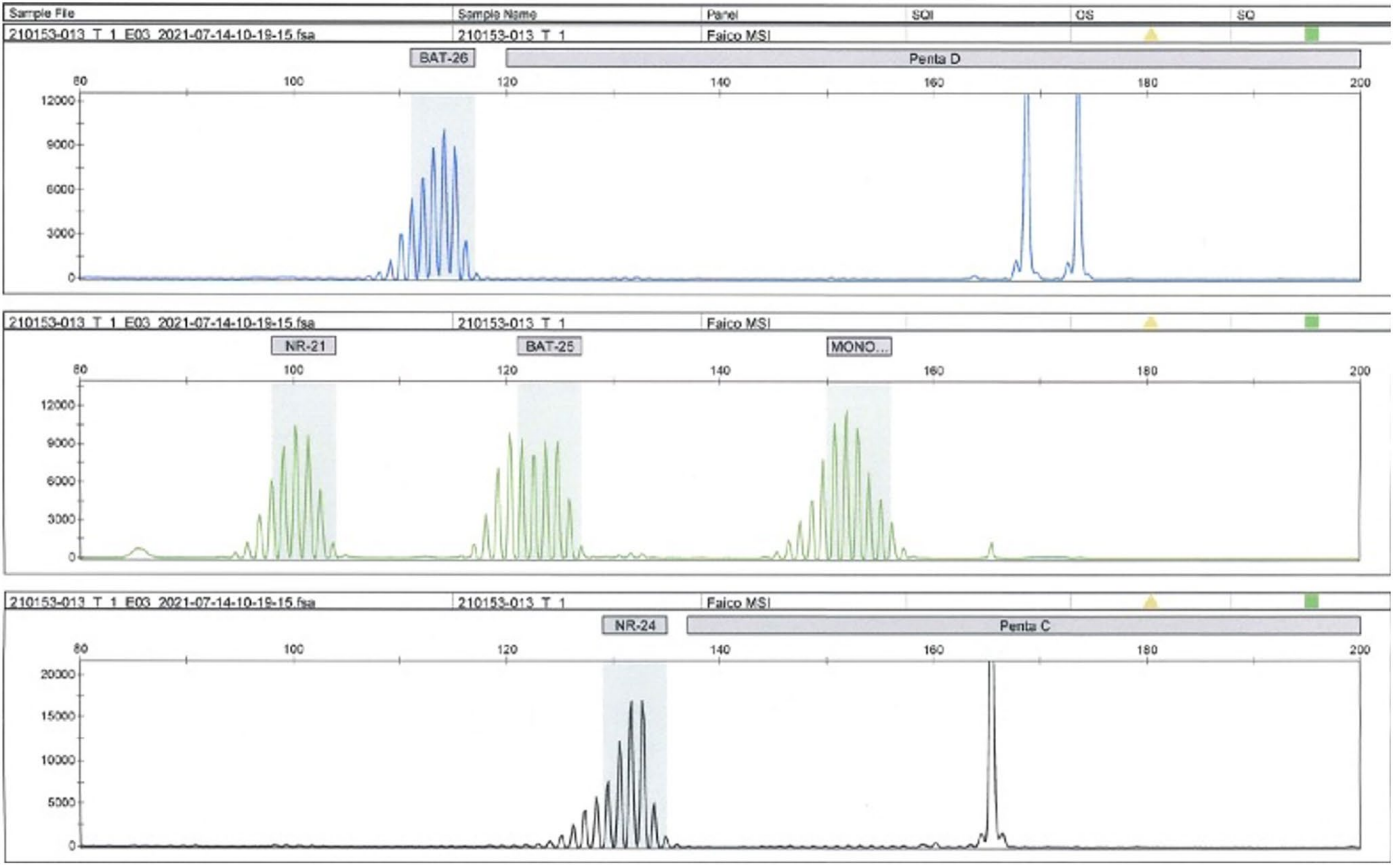
The electrophoresis figure of the microsatellite instability (MSI) assay. Five mononucleotide markers (BAT‐25, BAT‐26, MONO‐27, NR‐21, and NR‐24) dislocated from the standard area (gray area). Two pentanucleotide markers (PentaC and PentaD) are also displayed.

**FIGURE 5:**
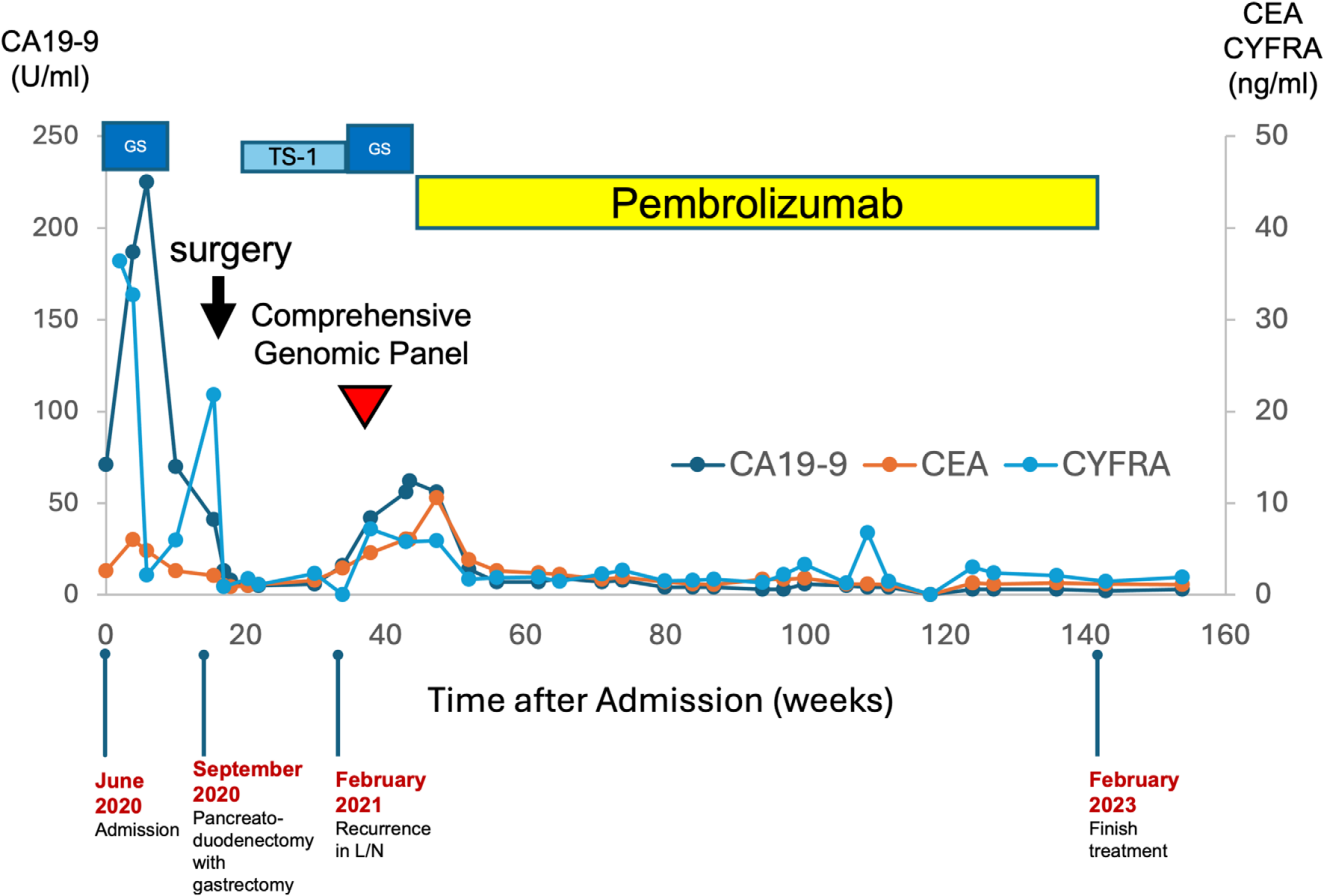
Clinical course of the patient in relation to tumor markers and the treatments. The chemotherapies administered are shown below: TS‐1 (tegafur/gimeracil/oteracil) and GS (gemcitabine and TS‐1).

A 72‐year‐old woman was admitted to Asahikawa Medical University Hospital with epigastric pain and underwent esophagogastroduodenoscopy (EGD) in June 2020. A duodenal tumor and gastric ulceration were discovered, and histological evaluation of the biopsy specimen indicated poorly differentiated adenocarcinoma and benign inflammation, respectively. She had a medical history of total hysterectomy for uterine myoma in her 40s and a family history of hepatocellular carcinoma in her brother. The Eastern Cooperative Oncology Group (ECOG) performance status was 0. Immunohistochemistry (IHC) was positive for CAM5.2, cytokeratin 7 (CK7), caudal type homeobox 2 (CDX2), and carcinoembryonic antigen (CEA), and negative for thyroid transcription factor‐1 (TTF‐1) and cytokeratin 20 (CK20), indicating pancreaticobiliary malignancy. CT showed an extra‐duodenal mass growing to the common bile duct and the gall bladder without distal metastasis to other organs (Figure 1a). Colonoscopy showed no colorectal tumors except for a small (4 mm) adenoma in the rectum. Laboratory investigations showed anemia (white blood cells, 5.56 × 10^3^/mm^3^; red blood cells, 2.68 × 10^3^/mm^3^, platelets 30.8 × 10^3^/mm^3^), slightly elevated lactose dehydrogenase (344 U/L; normal level 105–210 U/L), and tumor marker levels of 71 U/mL of Carbohydrate antigen 19‐9 (CA19‐9, normal level ≦ 37 U/mL), 2.6 ng/mL of CEA (normal level ≦ 5.0 ng/mL), and 36.4 ng/mL of cytokeratin 19 fragment (CYFRA, normal level ≦ 3.5 ng/mL). Endoscopic ultrasound fine‐needle biopsy of the tumor located in the pancreaticobiliary groove revealed poorly differentiated adenocarcinoma. Re‐examination with EGD revealed a laterally spreading gastric malignancy at the gastric angle, and a histological examination revealed well‐differentiated adenocarcinoma. The final diagnosis was dual cancer of unknown origin of pancreaticobiliary cancer and early gastric cancer. Gemcitabine and tegafur/gimeracil/oteracil (TS‐1) were chosen as neoadjuvant chemotherapy regimens, followed by pancreatoduodenectomy with distal gastrectomy (Figure 1b). Histological examination of the surgically resected specimens revealed poorly differentiated adenocarcinoma [T4a, N1, M1, pStage IVB], possibly of gallbladder origin, consistent with CUP, and well‐differentiated adenocarcinoma [T1b (SM2), N0, M0, pStage IA] of the stomach (Figure 2). The origin of CUP was suspected from biliary tract and adjuvant chemotherapy with TS‐1 was initiated; however, lymph node metastases around superior mesenteric artery (SMA) progressed 5 months later (Figure 3a). Gemcitabine with TS‐1 therapy was re‐attempted as the next regimen and a comprehensive cancer genomic panel (FoundationOne CDx, Foundation medicine, MA) was performed. Using formalin‐fixed paraffin‐embedded samples from CUP, many actionable genetic changes were found, and the microsatellite status was found to be high with a high tumor mutational burden (26.48 mutations/Mb). The analysis identified many actionable changes; *ARID1A* P225fs*175 (28.1%), *ERRFL1* P339fs*111 (29.6%), *KRAS* G12D (46.2%), *APC* S1465fs*3 (50.2%), *ATR* I774fs*3 (24.7%), *CDKN2A/B* S7fs*19 (28.7%), *CREBBP* P1946fs30 (32.4%), *FAM46C* A232T (31.9%), *FLCN* H429fs*39 (32.1%), *MLH1* A681T (2.2%), *MLL2* P647fs*283 (53.4%) P648fs* (21.2%), *MSH3* K383fs*32 (35.2%), *QKI* K134fs*14 (63.9%), *RICTOR* N1065S (51.2%), *TP53* R273C (64.2%), and *WHSC1* E1344fs*91 (31.6%). Furthermore, microsatellite instability (MSI) was confirmed using an MSI assay kit (FALCO Biosystems, Kyoto, Japan), and peak shifts of the five markers were found by capillary electrophoresis (Figure 4). These genetic findings suggested that CUP was a microsatellite unstable solid tumor; therefore, ICI was chosen as the next treatment for the recurrent lymph node metastasis. Pembrolizumab monotherapy (200 mg/body every 3 weeks) was administered. Lymph node metastasis shrank, and complete remission was maintained with no adverse event. Tumor marker levels of CA19‐9, CEA, and CYFRA were within normal range, EGD and colonoscopy presented no remnant cancers in the gastrointestinal tract. The treatment was stopped after 31 cycles (Figure 3b) and no recurrence was observed at 1 year after the cessation of treatment (Figure 5).

**FIGURE 6:**
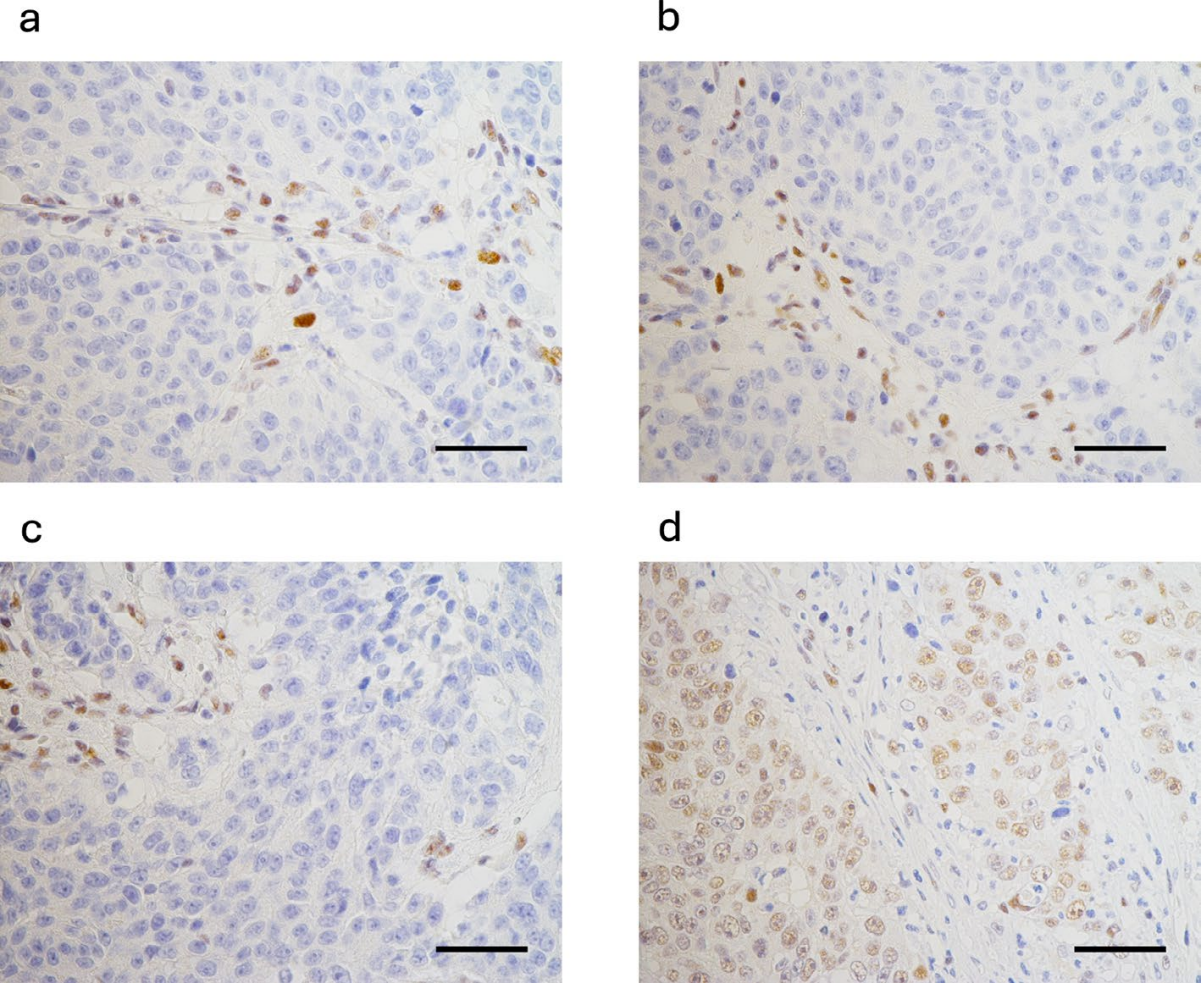
Immunohistochemical staining of mismatch repair (MMR) protein in the cancer of unknown primary. The expression of MLH1 (a), PMS2 (b), and MSH2 (c) in cancer cells is lost, while that of MSH6 (d) is sustained. The results indicate that the cancer is microsatellite instable. Scale bars: 50 μm.

As a result of the cancer genomic panel, *FLCN* H429fs*39 and *MLH1* A681T were suspected to be germline pathological variants (PGPV), and germline sequences of *FLCN* chr17: 17216395 and *MLH1* chr3: 37048955 were assessed by NGS (Kazusa DNA Research Institute, Chiba, Japan). No germline variants were detected in this study. An MLH1 variant is associated with Lynch syndrome due to the MSI‐high status; eventually, the result excluded the possibility of Lynch syndrome. Her family history did not meet the Lynch‐Amsterdam criteria either. IHC of mismatch repair (MMR) protein, M1 (Roche Diagnostics, Basel, Switzerland), G210‐1129 (Roche Diagnostics), PU29 (Leica Microsystems), and EP52 (Agilent Technology, Santa Clara, CA) were used for MutL homolog 1 (MLH1), MutS homolog 2 (MSH2), MSH6, and postmeiotic segregation increased 2 (PMS2) staining, respectively. The expression of MLH1, MSH2, and PMS2 was lost in the carcinoma cells of CUP (Figure 6).

**FIGURE 7:**
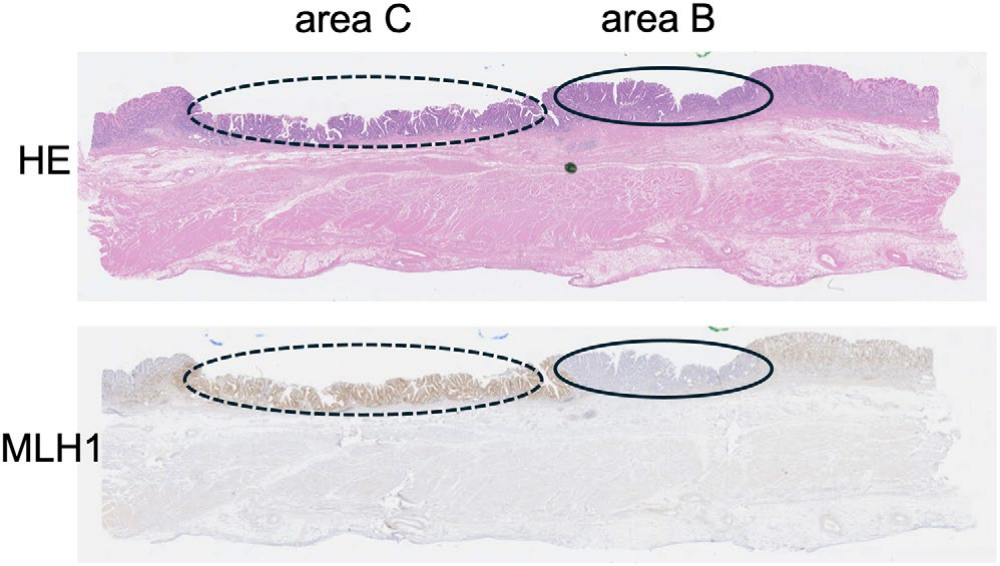
Immunohistochemical staining of the gastric cancer. Hematoxylin eosin (HE) staining shows a superficial tumor. Immunohistochemical staining of MLH1 shows different staining patterns: negative in area B (closed circle) and positive in area C (dotted circle). The samples from each area were subjected to a genetic analysis.

The CGP of the CUP tissue identified several pathogenic variants, including *TP53*, *KRAS*, and *APC*, which are frequently observed in gastrointestinal cancers. To further investigate the tumor origin, both the early gastric cancer and CUP specimens underwent IHC and target gene sequencing [8]. The well‐differentiated tubular adenocarcinoma showed a partial loss of MLH1, MSH2, and PMS2. Microdissection from each MLH1‐negative (area B) and MLH1‐positive area (area C) was conducted, and the samples were subjected to genetic testing (Figure 7 and Table 1). Both MLH1‐positive and ‐negative areas showed the same variations (i.e., *KRAS* G12D and *APC* S1465Wfs*3), indicating that these carcinomas share a common genotype. Eventually, the CUP was revealed from the origin of the gastric cancer cells.

**TABLE 1:** Somatic gene variants in different area of the cancer.

Area	Histology	Tissue	MLH1 IHC	Mean depth	Uniformity	Target base coverage at 100× (%)	Target base coverage at 500× (%)	*KRAS*			*APC*		
								Protein	Coding	Frequency (%)	Protein	Coding	Frequency (%)
A	Poorly differentiated adenocarcinoma	CUP	Negative	4010	95.5%	99.1%	97.3%	G12D	c.35G > A	54.6%	S1465WfsTer3	c.4393_4394delAG	58.9%
B	Well differentiated adenocarcinoma	Stomach	Negative	3977	96.3%	99.1%	97.4%	G12D	c.35G > A	28.0%	S1465WfsTer3	c.4393_4394delAG	36.3%
C	Well differentiated adenocarcinoma	Stomach	Positive	4024	95.4%	98.7%	97.3%	G12D	c.35G > A	31.8%	S1465WfsTer3	c.4393_4394delAG	43.4%
D	Normal reference	Duodenum	Positive	4354	95.9%	99.0%	97.7%	N.D.			N.D.		

Abbreviations: CUP, cancer of unknown primary; IHC, immunohistochemistry; MLH1, MutL homolog 1; N.D.: not detected.

## 3 Discussion

The primary origin of this case was not primarily identified due to the large duodenal tumor mass at the porta hepatis. No primary tumor was larger than the mass. A few endoscopic and histological examinations revealed a gastric tumor, but it was suspected to be a coincidentally occurring gastric cancer. Since the depth of the laterally spreading cancer was expected to be the submucosa, it was diagnosed as an early‐stage cancer. Initially, we planned to treat the CUP with neoadjuvant chemotherapy. Pancreatoduodenectomy with distal gastrectomy and cholecystectomy was performed to resect the CUP tumor and early gastric cancer together. Histologically poorly differentiated and well‐ to moderately differentiated adenocarcinoma were the final diagnoses of the CUP tumor and gastric tumor, respectively. Metastatic tumors usually have a poorly differentiated phenotype and sometimes undergo genetic changes after anti‐cancer chemotherapy. We hypothesized that gastric malignant cells might cause greater metastasis to the portal hepatis with invasion into the duodenum, gallbladder, and bile duct. Genetic testing using both CGP and target sequencing was useful for identifying the origin of the CUP in our case (Table 2).

**TABLE 2:** A summary of assessments.

Assessment	Time (weeks)	Area^a^	Findings	Diagnosis
Clinical	0	A	EUS‐FNA revealed poorly differentiated adenocarcinoma in the pancreaticobiliary groove	Unknown origin of pancreaticobiliary cancer
		B, C	EGD revealed a laterally spreading gastric malignancy at the gastric angle and a histological examination revealed well‐differentiated adenocarcinoma	Early gastric cancer
Pathological	20	A	Poorly differentiated adenocarcinoma, T4a, N1, M1, pStage IVB	Possibly of gallbladder origin
		B, C	Well‐differentiated adenocarcinoma, T1b (SM2), N0, M0, and pStage IA	Gastric cancer
Molecular	40	A	TMB‐high and MSI‐high; *ARID1A* P225fs*175, *ERRFL1* P339fs*111, *KRAS* G12D, *APC* S1465fs*3, *ATR* I774fs*3, *CDKN2A*/*B* S7fs*19, *CREBBP* P1946fs30, *FAM46C* A232T, *FLCN* H429fs*39, *MLH1* A681T, *MLL2* P647fs*283 P648fs*, *MSH3* K383fs*32, *QKI* K134fs*14, *RICTOR* N1065S, *TP53* R273C, and *WHSC1* E1344fs*91 The expression of MLH1, MSH2, and PMS2 was lost	MSI‐high tumor originated from gastric cancer
		B, C	A partial loss of MLH1, MSH2, and PMS2 Both MLH1‐positive and ‐negative areas showed the same variations (i.e., *KRAS* G12D and *APC* S1465Wfs*3)	Gastric cancer with same variations

^a^ Areas A–C are depicted in Table 1.

Some guidelines are available for the diagnosis and treatment. IHC analysis, which is used to analyze protein expression patterns, may provide a better understanding of the original carcinoma in the case with CUP [9, 10]. For instance, certain markers are associated with specific cancers (e.g., NKX3.1 for prostate cancer or SATB2 for colorectal cancer). Many IHC markers are recommended to indicate the primary cancer [9]. However, IHC is often not definitive, particularly in poorly differentiated or undifferentiated tumors. Therefore, genetic testing in the diagnosis of CUP is challenging. Advances in molecular diagnostics are expected to improve the ability to identify the primary sites of CUP. CGP allows for a comprehensive analysis of both DNA and RNA from tumor samples [11]. It can be used to identify actionable mutations and genetic alterations that may suggest the origin of the cancer. However, although NGS can detect potential therapeutic targets, it does not always provide a definitive primary site [7]. Treatment options for CUP are determined by clinical, pathological, and molecular genetic testing. Site‐directed therapy was decided according to the guidelines. If available, biomarker‐driven therapy can be chosen (e.g., *BRAF* V600E, *NTRK* gene fusion, MSI‐high, or HER2‐positive), which is expected to improve the patient prognosis. A comprehensive, multidisciplinary approach is essential, both for an accurate diagnosis and effective treatment planning. Advances in molecular biology and imaging techniques hold promise for improving the diagnostic accuracy and management of CUP.

Genetic alterations in gastric cancers have been well studied, and the variant frequencies were obtained from public databases. According to the cBioPortal website (Figure S1), *KRAS* and *APC* variants occur in 15% and 12% of stomach adenocarcinoma, respectively. In colorectal cancer, the same database reports 40% of *KRAS* and 65% of *APC* variants. Pancreatic cancer shows 83% of *KRAS* and 2% of *APC*, whereas gallbladder cancer shows frequencies of 10% and 3%, respectively. These data suggest colorectal cancer as a likely origin; however, total colonoscopic examination revealed no evidence of colorectal malignancy. Concurrent KRAS and APC variants are uncommon in gastric cancer. Therefore, a combination of targeted sequencing is useful for identifying cancers. CGP using CUP material showed variations in *KRAS* G12D and *APC* S1465Wfs*3 followed by the target NGS analysis. In particular, the *APC* variant S1465W was not coincidental because only 10 gastric cancer patients with the single nucleotide variation were deposited in cBioPortal (Figure S2). Therefore, CUP must be derived from gastric carcinoma. Tumor progression was suspected to have occurred as follows: The gastric tumor remained superficial within the original gastric tissue, while the metastatic cancer cells in the porta hepatis exhibited poor histology and rapid growth. Over time, the gastric tumor partially lost its MMR protein expression, and the metastatic tumor showed MSI‐high status. Gastric cancer develops into an MSI‐high genotype [12]. The recurrence of lymph node metastasis was eliminated by pembrolizumab treatment. As demonstrated in the KEYNOTE‐158 phase II multicohort study, a meaningful and durable benefit with a long‐term response to pembrolizumab is expected against MSI‐high cancers [13, 14, 15]. MSI‐high cancers have been reported in 1.6%–1.8% of CUP cases, and the efficacy of ICI treatment in this setting remains insufficiently studied [16, 17]. Kato et al. [18] reported a successful response to a combination of nivolumab and trametinib in a CUP patient harboring *MLH1* and *KRAS* mutations. Since CUP cases with MSI‐high or deficient MMR are rare and ICI administration in such cases has seldom been documented, our case—demonstrating a favorable ICI response—may be of particular clinical relevance. PD‐L1 status has been reported as a predictor of overall survival in CUP patients treated with ICI [17]. Further studies are warranted to identify predictive biomarkers for ICI sensitivity and improved survival.

A case with a large mass at the portal hepatis was primarily diagnosed as CUP, and a comprehensive approach identified it as a gastric cancer origin. Both the genetic testing and the genotype‐matched treatment with ICI successfully induced complete remission of metastatic cancer.

## Author Contributions

T.S. designed this case report and performed a whole study. S.Y. and M.T. contributed to histological diagnosis and immunohistochemical study. H.T. contributed to write this manuscript. Y.O. and Y.M. performed genetic analysis. K.T., K.A., N.U., and S.K. were involved in the patient's diagnosis and treatment. K.M. processed the experimental data and performed the analysis. M.F. supervises this research.

## Ethics Statement

The genetic analysis in the present case was approved by Asahikawa Medical University Hospital Ethical Committee (approval number: 19067).

## Consent

Informed consent for participation and publication was obtained from the patient.

## Conflicts of Interest

Y.O. and Y.M. received research funding from Hitachi High‐Tech Corporation.

## Supporting information


**Data S1:** Supporting Information.

## Data Availability

The data that support the findings of this study are available on request from the corresponding author. The data are not publicly available due to privacy or ethical restrictions.

## References

[1] A. Krämer , T. Bochtler , C. Pauli , et al., “Cancer of Unknown Primary: ESMO Clinical Practice Guideline for Diagnosis, Treatment and Follow‐Up,” Annals of Oncology 34 (2023): 228–246, 10.1016/j.annonc.2022.11.013. 36563965

[2] E. Rassy , S. Boussios , and N. Pavlidis , “Genomic Correlates of Response and Resistance to Immune Checkpoint Inhibitors in Carcinomas of Unknown Primary,” European Journal of Clinical Investigation 51 (2021): e13583, 10.1111/eci.13583. 33970501

[3] B. Yu , Q. Wang , X. Liu , et al., “Case Report: Molecular Profiling Assists in the Diagnosis and Treatment of Cancer of Unknown Primary,” Frontiers in Oncology 12 (2022): 723140, 10.3389/fonc.2022.723140. 35433426 PMC9005951

[4] J. Sheng , H. Pan , and W. Han , “Immunochemotherapy Achieved a Complete Response for Metastatic Adenocarcinoma of Unknown Primary Based on Gene Expression Profiling: A Case Report and Review of the Literature,” Frontiers in Immunology 14 (2023): 1181444, 10.3389/fimmu.2023.1181444. 37153561 PMC10154565

[5] PDQ Adult Treatment Editorial Board , “Cancer of Unknown Primary (CUP) Treatment (PDQ): Health Professional Version,” in PDQ Cancer Information Summaries (National Cancer Institute (US), 2024).

[6] PDQ Adult Treatment Editorial Board , Cancer of Unknown Primary (CUP) Treatment (PDQ), https://www.ncbi.nlm.nih.gov/books/NBK65811/.

[7] L. Boscolo Bielo , C. Belli , E. Crimini , et al., “Cancers of Unknown Primary Origin: Real‐World Clinical Outcomes and Genomic Analysis at the European Institute of Oncology,” Oncologist 29 (2024): 504–510, 10.1093/oncolo/oyae038. 38520742 PMC11145013

[8] H. Tanabe , K. Moriichi , K. Takahashi , et al., “Genetic Alteration of Colorectal Adenoma‐Carcinoma Sequence Among Gastric Adenocarcinoma and Dysplastic Lesions in a Patient With Attenuated Familial Adenomatous Polyposis,” Molecular Genetics & Genomic Medicine 8 (2020): e1348, 10.1002/mgg3.1348. 32543103 PMC7507424

[9] P. Krawczyk , J. Jassem , K. Wojas‐Krawczyk , M. Krzakowski , R. Dziadziuszko , and W. Olszewski , “New Genetic Technologies in Diagnosis and Treatment of Cancer of Unknown Primary,” Cancers 14 (2022): 3429, 10.3390/cancers14143429. 35884492 PMC9318615

[10] A. Qaseem , N. Usman , J. S. Jayaraj , R. N. Janapala , and T. Kashif , “Cancer of Unknown Primary: A Review on Clinical Guidelines in the Development and Targeted Management of Patients With the Unknown Primary Site,” Cureus 11 (2019): e5552, 10.7759/cureus.5552. 31695975 PMC6820325

[11] Y. Zhang , L. Xia , D. Ma , J. Wu , X. Xu , and Y. Xu , “90‐Gene Expression Profiling for Tissue Origin Diagnosis of Cancer of Unknown Primary,” Frontiers in Oncology 11 (2021): 722808, 10.3389/fonc.2021.722808. 34692498 PMC8529103

[12] H. Tanabe , Y. Mizukami , H. Takei , et al., “Clinicopathological Characteristics of Epstein‐Barr Virus and Microsatellite Instability Subtypes of Early Gastric Neoplasms Classified by the Japanese and the World Health Organization Criteria,” Journal of Pathology. Clinical Research 7 (2021): 397–409, 10.1002/cjp2.209. 33750036 PMC8185367

[13] A. Marabelle , D. T. Le , P. A. Ascierto , et al., “Efficacy of Pembrolizumab in Patients With Noncolorectal High Microsatellite Instability/Mismatch Repair‐Deficient Cancer: Results From the Phase II KEYNOTE‐158 Study,” Journal of Clinical Oncology 38 (2020): 1–10, 10.1200/JCO.19.02105. 31682550 PMC8184060

[14] M. Maio , P. A. Ascierto , L. Manzyuk , et al., “Pembrolizumab in Microsatellite Instability High or Mismatch Repair Deficient Cancers: Updated Analysis From the Phase II KEYNOTE‐158 Study,” Annals of Oncology 33 (2022): 929–938, 10.1016/j.annonc.2022.05.519. 35680043

[15] K. P. Raghav , B. Stephen , D. D. Karp , et al., “Efficacy of Pembrolizumab in Patients With Advanced Cancer of Unknown Primary (CUP): A Phase 2 Non‐Randomized Clinical Trial,” Journal for Immunotherapy of Cancer 10 (2022): e004822, 10.1136/jitc-2022-004822. 35618285 PMC9125753

[16] Z. Gatalica , J. Xiu , J. Swensen , and S. Vranic , “Comprehensive Analysis of Cancers of Unknown Primary for the Biomarkers of Response to Immune Checkpoint Blockade Therapy,” European Journal of Cancer 94 (2018): 179–186, 10.1016/j.ejca.2018.02.021. 29571084

[17] J. N. A. Junior , D. D. Preto , M. E. Z. N. Lazarini , et al., “PD‐L1 Expression and Microsatellite Instability (MSI) in Cancer of Unknown Primary Site,” International Journal of Clinical Oncology 29 (2024): 726–734, 10.1007/s10147-024-02494-3. 38528294 PMC11130030

[18] S. Kato , N. Krishnamurthy , K. C. Banks , et al., “Utility of Genomic Analysis in Circulating Tumor DNA From Patients With Carcinoma of Unknown Primary,” Cancer Research 77 (2017): 4238–4246, 10.1158/0008-5472.CAN-17-0628. 28642281 PMC5729906

