# Supplementary material for: Successful Identification and Treatment of Cancer of Unknown Primary Originating From Gastric Cancer Using Comprehensive Genomic Profiling and Immune Checkpoint Inhibitor Therapy: A Case Report
Source: Cancer Rep (Hoboken). 2025 Sep 8;8(9):e70338. doi: 10.1002/cnr2.70338 (PMC12415352; doi:10.1002/cnr2.70338)
Supplement: Supplementary file 1 — Data S1: Supporting Information. [file CNR2-8-e70338-s001.docx]

**Supplementary Figures**

**Figure S1**

**a**

In stomach adenocarcinoma, 15% of *KRAS* and 12% of *APC* variants were observed in the cBioPortal website (https://www.cbioportal.org/results/oncoprint?tab_index=tab_visualize&Action=Submit&session_id=66dc0eb883e9543d6191eb8c&plots_horz_selection=%7B%7D&plots_vert_selection=%7B%7D&plots_coloring_selection=%7B%7D&mutations_gene=APC).

**b**

In colorectal cancer, 40% of *KRAS* and 65% of *APC* variants were observed.

**c**

In pancreatic adenocarcinoma, 83% of *KRAS* and 2% of *APC* variants were observed.

**d**

In gallbladder cancer, 19% of *KRAS* and 3% of *APC* variants were observed.

**Figure S2**

a

*KRAS* variants are concentrated in codon 12 and G12D is one of the most frequent single nucleotide variants indicated by arrow.

b

*APC* variants are distributed in the entire gene. S1465Efs*3 indicated by arow head shows 10 patients are deposited in the cBioPortal.
